# Deciphering the Morphology of Motor Evoked Potentials

**DOI:** 10.3389/fninf.2020.00028

**Published:** 2020-07-14

**Authors:** Jan Yperman, Thijs Becker, Dirk Valkenborg, Niels Hellings, Melissa Cambron, Dominique Dive, Guy Laureys, Veronica Popescu, Bart Van Wijmeersch, Liesbet M. Peeters

**Affiliations:** ^1^Theoretical Physics, Hasselt University, Diepenbeek, Belgium; ^2^I-Biostat, Data Science Institute, Hasselt University, Diepenbeek, Belgium; ^3^BIOMED, Hasselt University, Diepenbeek, Belgium; ^4^Faculty of Medicine and Pharmacy, Vrije Universiteit Brussel (VUB), Brussels, Belgium; ^5^Department of Neurology, AZ Sint-Jan, Brugge, Belgium; ^6^Neurology, CHU Liège, Esneux, Belgium; ^7^Department of Neurology, University Hospital Ghent, Ghent, Belgium; ^8^Revalidation and MS Center Pelt, Pelt, Belgium

**Keywords:** motor evoked potentials, morphology, multiple sclerosis, machine learning, approximate entropy

## Abstract

Motor Evoked Potentials (MEPs) are used to monitor disability progression in multiple sclerosis (MS). Their morphology plays an important role in this process. Currently, however, there is no clear definition of what constitutes a normal or abnormal morphology. To address this, five experts independently labeled the morphology (normal or abnormal) of the same set of 1,000 MEPs. The intra- and inter-rater agreement between the experts indicates they agree on the concept of morphology, but differ in their choice of threshold between normal and abnormal morphology. We subsequently performed an automated extraction of 5,943 time series features from the MEPs to identify a valid proxy for morphology, based on the provided labels. To do this, we compared the cross-validation performances of one-dimensional logistic regression models fitted to each of the features individually. We find that the approximate entropy (ApEn) feature can accurately reproduce the majority-vote labels. The performance of this feature is evaluated on an independent test set by comparing to the majority vote of the neurologists, obtaining an AUC score of 0.92. The model slightly outperforms the average neurologist at reproducing the neurologists consensus-vote labels. We can conclude that MEP morphology can be consistently defined by pooling the interpretations from multiple neurologists and that ApEn is a valid continuous score for this. Having an objective and reproducible MEP morphological abnormality score will allow researchers to include this feature in their models, without manual annotation becoming a bottleneck. This is crucial for large-scale, multi-center datasets. An exploratory analysis on a large single-center dataset shows that ApEn is potentially clinically useful. Introducing an automated, objective, and reproducible definition of morphology could help overcome some of the barriers that are currently obstructing broad adoption of evoked potentials in daily care and patient follow-up, such as standardization of measurements between different centers, and formulating guidelines for clinical use.

## 1. Introduction

Multiple sclerosis (MS) is characterized by disruption of electrical signal conduction over axons in the central nervous system by a variety of mechanisms, including the loss of the myelin sheath (Emerson, 1998). Evoked potential (EP) disturbances have been widely utilized in people with MS (PwMS) to demonstrate the involvement of sensory, visual, auditory, and motor pathways. The advent of magnetic resonance imaging (MRI) techniques has greatly reduced the clinical utilization of EPs, which is not fully justifiable, as the information provided by EPs is quite different from that provided by MRI. The abnormalities of evoked responses reflect the global damage of the evoked nervous pathway and are significantly correlated with the clinical symptoms, while the vast majority of MRI lesions are not (Comi et al., 1999). As such EPs are a functional counterpart to the anatomical findings on MRI.

The diagnostic value of EPs is based on the ability to reveal clinically silent lesions and to objectivate the central nervous system damage in PwMS, who complain of vague and indefinite disturbances which frequently occur in the early phases of the disease (Comi et al., 1999). Besides their diagnostic value, EPs may serve as useful instruments for assessing the effectiveness of therapeutic agents which may alter the course of the MS. The availability of new treatments able to modify the natural course of MS has generated interest in paraclinical measures like EPs to monitor disease evolution. Furthermore, since EPs measure conduction within the central nervous system, they provide a means of directly assessing symptomatic treatments designed to improve central conduction (Emerson, 1998). Finally, several recent findings demonstrate the utility of EP for predicting the course of the disease in patients (Fraser et al., 2006; Kallmann et al., 2006; Jung et al., 2008; Invernizzi et al., 2011; Margaritella et al., 2012; Schlaeger et al., 2014; Giffroy et al., 2016; London et al., 2017). For these purposes, EPs show better potential than conventional MRI (Fuhr and Kappos, 2001).

EPs are time series, resulting in high-dimensional data. For example, the motor EP (MEP) studied in this work span 100 ms and are sampled at 19.2 kHz, so we end up with 1,920 measurement points (i.e., dimensions). To significantly lower their dimensionality and to capture their salient information, evoked potential time series (EPTS) are often condensed into a single EP score (Schlaeger et al., 2016). Recent work has also investigated in reducing the dimensionality of an EP by using principal component regression (Nguyen et al., 2019). The EP score is a composite score, for which three variables are commonly extracted from the EPTS: latency, amplitude, and presence of morphological abnormality (Schlaeger et al., 2016). The first two variables are clearly defined, and can therefore be extracted automatically. Morphology, in contrast, does not have a simple operational definition, and depends on the interpretation of the neurologist.

The lack of an objective and reproducible definition leads to several issues, both in the clinic and for research purposes. When scoring the morphological abnormality of an EP, how dependent is the result on the neurologist? While EPs contain valuable information about the disease course, they are currently suboptimally utilized in clinics as their interpretation varies between clinics and requires expert knowledge. A clear definition for the morphology negates the need for an EP expert and could facilitate a wider adoption of this marker. From a research point of view, if morphology scoring is moderately inconsistent, the resulting EP score is noisier and less suited for statistical modeling. If scoring is highly inconsistent, one can wonder if morphology is a sensitive and well-defined concept. Current studies on EPs use at most a few 100 EPTS, which can be annotated manually (Leocani et al., 2006; Invernizzi et al., 2011; Schlaeger et al., 2016; London et al., 2017). But what if the number of EPs is orders of magnitude larger? Letting neurologists manually annotate the morphology of such a large number of EPs is not practically feasible. Finally, while there is agreement that the latency, peak-to-peak amplitude, and morphology of an EP are of clinical interest, their precise usefulness is still under debate. An automated and standardized score for morphological abnormality will greatly aid investigating the clinical usefulness of EP morphology.

In this work, five neurologists independently assign a binary label on the morphological abnormality of 1,000 motor EPTS (MEPTS). We investigate to what extent their labels agree. We use a machine learning approach to show that a single variable extracted from the EPTS, namely approximate entropy (ApEn), is able to reproduce the morphology classification to a high degree of fidelity. A graphical explanation of what ApEn measures in MEPTS is provided. Finally, we perform an exploratory analysis on its possible clinical usefulness on a real-world dataset.

## 2. Materials and Methods

### 2.1. Description of the Dataset

**Figure 1:**
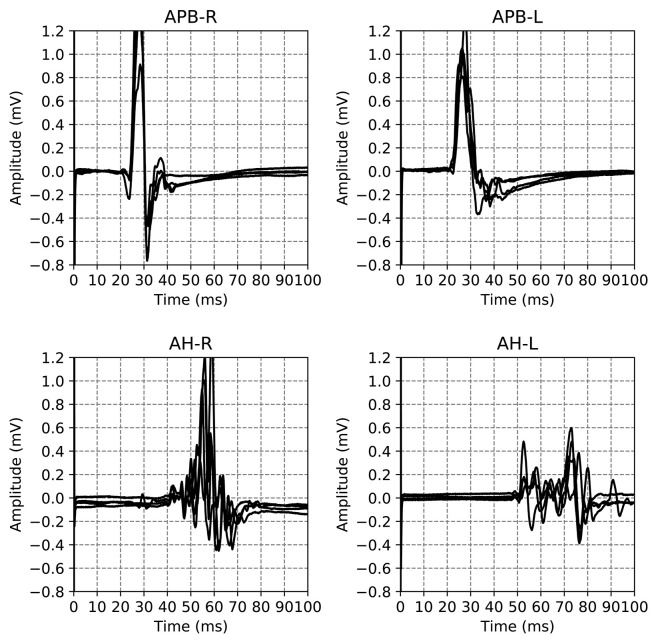
Example of the motor evoked potential time series recorded at a single hospital visit. The labels on the plot indicate the limb and the side on which the measurement was performed, M. Abductor Pollicis Brevis (APB) for the hands, M. Abductor Hallucis (AH) for the feet. The sides are indicated using R and L for right and left respectively. The time series for the same limb are the result of different magnetic excitation strengths. Adapted from Yperman et al. (2020).

The dataset used in this work is a subset of a retrospective dataset of full MEPTS that were collected in standard longitudinal follow-up at the Revalidatie en MS Centrum (RMSC) in Pelt, Belgium. A visit consists of two hands [M. abductor pollicis brevis (APB)] and 2 feet [M. abductor hallucis (AH)] measurements. An example of the set of measurements made in a single visit is shown in Figure 1. From this dataset we selected 225 visits (each containing 4 MEPs) at random. Table 1 shows the descriptive statistics of the cohort used in this study.

**Table 1:** Summary of the descriptive statistics of the cohort used in this study.

**MS type**	**No. of patients**	**Age (SD)**	**EDSS (SD)**	**F/M**	**No. of visits**
Unknown	29	44 (15)	2.8 (2.0)	21/8	34
PPMS	6	57 (7)	3.7 (1.3)	4/2	7
RRMS	107	43 (11)	2.4 (1.3)	82/25	164
SPMS	13	54 (8)	4.7 (1.6)	7/6	20
All	155	45 (12)	2.8 (1.6)	114/41	225

*PPMS, Primary progressive MS; RRMS, Relapsing-remitting MS; SPMS, Secondary progressive MS*.

This study was approved by the ethical commission of the University of Hasselt (CME2017/729). No consent to participate/publish was necessary since this study uses retrospective data only.

### 2.2. Measurement Protocol

Motor evoked potentials were recorded from the abductor pollicis brevis and abductor hallucis muscles bilaterally. Magnetic stimuli were delivered to the hand and leg areas of the motor cortex with a Magstim 200^2^ device (The Magstim Company Ltd., Whitland, UK) via a round coil with an inner diameter of 9 cm with maximal output of the stimulator of 2.2 T. The signal is recorded for 100 ms. The acquisition rate is 19.2 or 20 kHz. The 20 kHz signals are down-sampled to 19.2 kHz. Recording is done with two different machines. Signals from one machine are filtered between 0.6 Hz and 10 kHz, while the other machine has a high-pass filter of 100 Hz. We discuss the impact of this difference in machine setting in section 7 of the Supplementary Materials. The measurements are not averaged across multiple trials.

The measurements are performed in a standardized way to minimize variations due to factors such as coil orientation, stimulus intensity etc. For the hands, electrodes are placed at three places: on top of the hand (ground), the APB muscle, and the proximal phalanx of the thumb. The first excitation is at 45% of the maximal stimulator output. New stimuli are presented with an increase of 5% points.

For the feet, electrodes are placed at three places: on top of the foot (ground), the big toe, and the AH muscle. The first excitation is at 50% of the maximal stimulater output. New stimuli are presented with an increase of 5% points.

The measurement ends if the amplitude stops increasing for stronger stimuli, as judged from the lack of increase in amplitude of a few consecutive single MEPs. If the patient is expected to have a large maximal amplitude (much larger than 1 millivolt), one stops the measurements if an amplitude of 1 millivolt is reached. If the signal is of bad quality, as judged by the nurse, it is discarded.

An example of all the EPTS of the MEP for a single visit is shown in Figure 1. For each limb, each excitation strength gives one EPTS. After discussion with the neurologists we decided to use only the EPTS with the maximal peak-to-peak amplitude, as this is likely to be the most informative measurement.

### 2.3. Consensus Building on the Clinical Definition of MEPTS Morphology: Workshops

Two workshops were organized via teleconference. Five MS neurologists from 4 different hospitals across Belgium participated. All neurologists had extensive expertise in using EPs in their clinic. The goal of the two workshops was to come to an agreement on how to label the morphology of a MEP. More specifically, each limb should be labeled as having either normal or abnormal morphology, or as “bad data.” If a time series is labeled as “bad data” by at least one of the neurologists, e.g., when a time series contains measurement artifacts, it is discarded.

A few randomly sampled visits were discussed in the first teleconference. Afterwards, 50 visits were labeled independently by each neurologist. In the second teleconference, visits with the most disagreement in assigned label were discussed, to clear up any possible differences in interpretation. After the second teleconference, 225 new visits were labeled. Twenty-five visits are labeled twice, to measure intra-rater variability.

The EPTS for all excitation strengths are visible when rating. However, only the EPTS with the maximal peak-to-peak amplitude is rated. By showing all EPTS, the rater can take the excitation strength into account for judging morphological abnormality.

### 2.4. Online Labeling and Definition of Ground Truths

Labeling was done using a web-based labeling tool (see the Supplementary Materials for details). Tools were provided to pan, zoom, and hide/show individual time series.

To evaluate the performance of any rater, be it an algorithm or a person, ground truth labels are required. For the morphology of a MEPTS, there is no objective ground truth (in contrast to, e.g., detecting the presence of a tumor on an MRI, which can be confirmed with a biopsy). Therefore, we use the majority vote of the annotators. Since there are 5 votes, there is a consensus for each MEPTS. We will refer to the ground truth labels obtained in this way as the **5-vote** labels. The labels of the individual neurologists are referred to as the **1-vote** labels.

**Figure 2:**
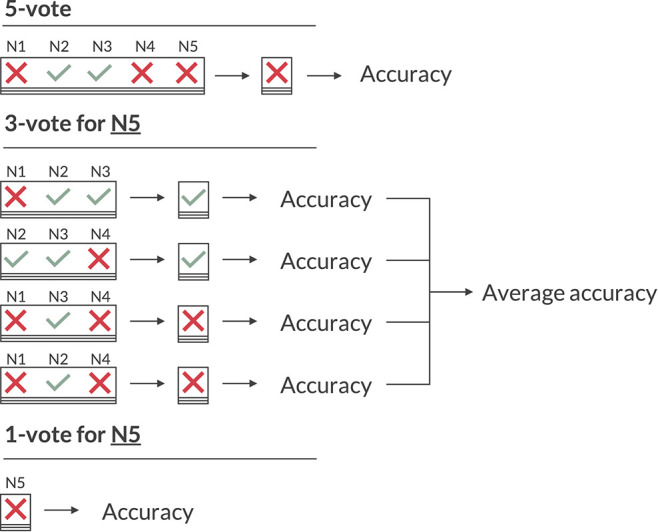
Illustration of the various voting schemes when calculating the accuracy. The 5-vote indicates how one would evaluate the model on the majority vote of the five neurologists (N1 to N5). The 3-vote illustrates how one would evaluate N5 (and similarly for N1-4). The 1-vote illustrates how one would evaluate the model on the labels of just one neurologist (in this case N5).

When evaluating the performance of the neurologists, however, we cannot use the 5-vote labels, as the label of the neurologist that is to be evaluated is included in that vote, skewing any performance metric in their favor. Just omitting their annotations isn't ideal either, as with four labels some MEPTS don't have a consensus (two against two votes). Therefore, we opt for having 4 sets of ground truths per neurologist, created by leaving out each of the remaining four neurologists once. These ground truth labels will be referred to as the **3-vote** labels. Any performance metric based on the 3-vote labels is the average across the four sets. This is illustrated for neurologist 5 (N5) in Figure 2.

As the 3-vote labels are less stable than those of the 5-vote, we evaluate our model on each of these 20 (5 neurologists × 4 sets of ground truths) sets as well, and average any performance metric across them. An illustration of how the 5-, 3-, and 1-vote are calculated for the accuracy measure is shown in Figure 2.

### 2.5. Data Analysis

5,943 features from different time series analysis methodologies are extracted from the MEPTS with the highly comparative time-series analysis (HCTSA) package (Fulcher et al., 2013; Fulcher and Jones, 2017). A wide variety of features is calculated in HCTSA, ranging from simple ones such as the mean of the time series, to more complex ones such as the average error of an autoregressive model that predicts the next point in the time series. Details of the HCTSA computation are the same as described in Yperman et al. (2020).

The dataset is divided into a training and test set, 50% train and 50% test, for which we ensure that there is no overlap in the patients. First of all, we want to find a single time series feature that can be used as a proxy for the morphology of the time series. Our goal is not so much to maximize the classification performance of our model, but rather to find an interpretable way of automating the labeling process on the level of the average neurologist. To do this, we fit a one-dimensional logistic regression model to each of the 5,943 time series features. This is done on the labels of each individual neurologist separately (1-vote). We use the 1-vote labels instead of the 5-vote labels to avoid the thresholding problem, which is discussed in further detail in section 3.4. We use one-dimensional logistic regression as it allows for an easy interpretation of the result, since one simply determines a threshold value of a one-dimensional statistic. Using 3-fold cross-validation on the training set we rank all features by their average AUROC score (Area Under the Receiver Operating Characteristics curve), usually referred to as just AUC in the literature. We compare the resulting top 10 features of each neurologist to find any overlap between them. A common feature is picked to use as a proxy for the morphology. To evaluate the performance of said feature, we train a logistic regression model on only this feature, using the 5-vote and 3-vote labels as targets. For the performance metrics that require a binary label, we choose the threshold where the true positive rate minus the false positive rate is maximal for the complete training set (a.k.a. the Youden index method). Finally, we measure the performance of the model on the test set.

The code that implements this analysis, as well as the dataset, has been made available at https://github.com/JanYperman/deciphering-morphology.

### 2.6. Performance Metrics

To evaluate the inter- and intrarater reliability of the labeling process we calculate both the agreement fractions and the Cohen's Kappa coefficients (κ) (Cohen, 1960). The former indicates the fraction of time series for which the neurologists on average agree. As this metric is greatly influenced by class imbalance, we also calculated the Cohen's κ. The Cohen's κ corrects for this class imbalance, and is influenced equally by the agreement errors in both classes. Cohen's κ ranges from −1 to 1, with 1 being perfect agreement. If there is no agreement among the raters other than what would be expected by chance κ = 0. If agreement is worse than random κ < 0. Both the agreement fraction and the Cohen's κ for the inter-rater reliability are evaluated pairwise between the neurologists, meaning each of them is compared to the other four for a total of 20 inter-rater reliability scores. For the final result we take the average of these scores. The intra-rater reliability is evaluated on the 100 timeseries that were labeled twice by the experts, as discussed in section 2.1.

Performance of the model on the test set is evaluated using several classification performance metrics. Those *without* a choice of threshold: AUC and average precision. Those *with* a choice of threshold: F1-score, accuracy, precision, recall, and Cohen's κ. The F1-score is the harmonic mean of the precision and the recall: F1 = 2 (precision × recall)/(precision + recall). For the model we compute these for both the 5-vote labels and the average across the 3-vote label sets. For the neurologists, we compute these measures only across the 3-vote label sets, as the 5-vote labels results would be skewed, as discussed in section 2.4.

## 3. Results and Discussion

### 3.1. Inter- and Intra-Rater Agreement Between the Neurologists

In total 3.6% of the MEPTS are discarded because at least one neurologist labeled them to be bad data. Around 74% is labeled as normal, so there is a class imbalance. The inter- and intra-rater scores are summarized in Table 2. We show both the agreement fraction and the Cohen's kappa coefficient.

**Table 2:** The results of the inter- and intra-rater scores.

	**Agreement fraction**	**Cohen's kappa**
N1	0.85 (0.96)	0.64 (0.90)
N2	0.85 (0.90)	0.63 (0.76)
N3	0.82 (0.93)	0.59 (0.85)
N4	0.80 (0.91)	0.48 (0.67)
N5	0.74 (0.78)	0.45 (0.57)
Average	0.81 (0.90)	0.56 (0.75)

*The inter-rater score is the average of the neurologist's agreement with the others, as defined in section 3.1. The intra-rater score is shown in brackets. Both the agreement fraction and the Cohen's kappa scores are shown*.

We find there is good agreement between the neurologists. The average inter-rater (81%) and intra-rater (90%) agreement fractions are high. A judgment on the quality of the obtained Cohen's κ score is by definition subjective. Following the (often used) labeling from Landis and Koch (1977) (<0: Poor, 0–0.2: slight, 0.21–0.4: fair, 0.41–0.6: moderate, 0.61–0.8: substantial, 0.81–1.0: almost perfect), the inter-rater Cohen's κ of 0.56 is a moderate agreement level, and the intra-rater Cohen's κ of 0.75 is a substantial agreement level. However, any differences in labeling can be mostly attributed to the individual choice of threshold, which we discuss in detail in section 3.4. Therefore, we conclude that morphological abnormality is consistently rated. The obtained labels can therefore be used to create ground-truth datasets, which are the basis for automating the morphology extraction from MEPTS.

### 3.2. Selected Feature: Approximate Entropy, a Measure for Time Series Regularity

To select the feature that can serve as a proxy for morphology, we inspect the top 10s of the best performing features for the individual neurologists' classifications (1-votes). Approximate entropy (ApEn) occurs in the top 10 of each individual neurologist, with an average AUC of 0.92. This makes it a prime candidate for it to be used as a proxy for the experts' interpretation of the morphology. ApEn was originally introduced to quantify the regularity of a time series (Pincus and Goldberger, 1994). It is a dimensionless quantity which, in the case of a MEP, basically measures the strength and duration of its fluctuations. The interpretation of ApEn for MEPTS is discussed in section 3.5. The fact that it independently occurs in the top 10 of each expert corroborates the conclusion from the previous section that morphology is a consistently rated quantity. For a more detailed discussion of the top 10s, we refer the reader to section 1 of the Supplementary Materials.

### 3.3. Performance of Approximate Entropy

From a visual inspection of the performance on the 1-vote labels we have chosen ApEn as the morphology feature. To test whether it indeed works well, we evaluate its performance on the 3-vote and 5-vote labels, on an independent test set, using several metrics. These results are shown in Table 3. The performance of the logistic regression model with ApEn as its only input either exceeds or matches that of the average neurologist on unseen MEPTS, indicating that ApEn can be used effectively for scoring morphological abnormality. For the metrics that required binary labels, the approximate entropy threshold was chosen to be 0.545. For the performances of the model for each of the machines separately to study the impact of the filter, we refer the reader to section 7 of the Supplementary Materials.

**Table 3:** Summary of all performance metrics.

	**Model [5-vote] (std)**	**Model [3-vote] (std)**	**Neurologists [3-vote] (std)**
AUC	0.92 (0.01)	0.92 (0.01)	N/A
AP	0.85 (0.01)	0.82 (0.02)	N/A
F1	0.81 (0.01)	0.77 (0.02)	0.71 (0.08)
Accuracy	0.89 (0.01)	0.87 (0.02)	0.82 (0.05)
Precision	0.84 (0.01)	0.81 (0.03)	0.76 (0.17)
Recall	0.78 (0.02)	0.74 (0.05)	0.75 (0.17)
Cohen	0.73 (0.02)	0.68 (0.03)	0.60 (0.11)

*The values shown in brackets are the standard deviations of the average performance. Recall that the performance of each neurologist is averaged across 4 3-vote ground truth sets. For the 5-vote labels, we subsampled the test set 5 times (at 80%) to obtain the value for the standard deviation. Note that for these metrics the abnormal class was used as the positive label. AUC, Area Under the receiver operating characteristic Curve; AP, Average Precision*.

### 3.4. Individual Neurologists Differ in ApEn Threshold

Neurologists were asked to classify each MEPTS into two classes (normal vs. abnormal). However, the morphological abnormality of a MEPTS is not a binary characteristic, but a continuous score, revealing different degrees of morphological abnormality. Our dichotomization makes each neurologist choose a threshold at which the class goes from normal to abnormal. Because in our case the choice of this threshold is rather arbitrary, it will almost inevitably differ among neurologists. In this section, we show how a significant portion of the disagreement can be attributed to the choice of threshold.

**Figure 3:**
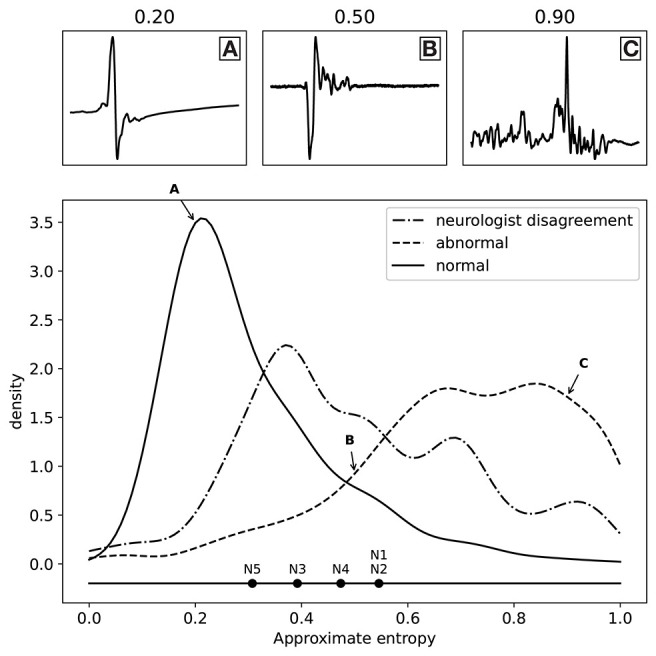
Distribution of the labels (normal and abnormal) as a function of approximate entropy, as well as the distribution of MEPTS (motor evoked potential time series) with the most disagreement among the neurologists (i.e., those where there is a 2 against 3 vote). The distributions are made using a Gaussian kernel of width 0.05. To illustrate what various values of approximate entropy look like, a few samples are shown, taken from the distribution at the locations indicated by the A, B, and C labels (approximate entropy equal to 0.2, 0.5, and 0.9, respectively). The individual thresholds of the experts are also shown to illustrate the thresholding problem.

As discussed above, we have fitted a logistic regression model for each neurologist separately to find the best possible proxy for morphology, resulting in five separate models. All these models worked very well when using the approximate entropy feature, which indicates that all the experts were annotating the same feature of the MEPTS, and that they differ mostly in their choice of threshold. We can illustrate this thresholding issue by estimating the threshold each neurologist has chosen on the approximate entropy scale. In the one-dimensional case, fitting a logistic regression model boils down to choosing a threshold above which everything is classified as abnormal, and everything below it is classified as normal. These thresholds can be interpreted as the thresholds of each of the neurologists. They are illustrated in Figure 3, from which it can be seen that they center around ~0.5, though there are clearly some differences. For example, N5 is quicker to label a time series as abnormal compared to the others, while N1 and N2 have very similar thresholds.

The distributions of the ApEn values for the normal and abnormal class are also shown in Figure 3. The two classes clearly lie on different sides. From the distribution of samples with a 2 against 3 vote, we see an increased disagreement rate of the classification of the neurologists for MEPTS with moderate ApEn. The individual thresholds again suggest that the increased disagreement rate of MEPTS with moderate ApEn is not so much due to a difference in interpretation of morphology, but rather an unfortunate side-effect of dichotomizing an inherently continuous measure.

**Figure 4:**
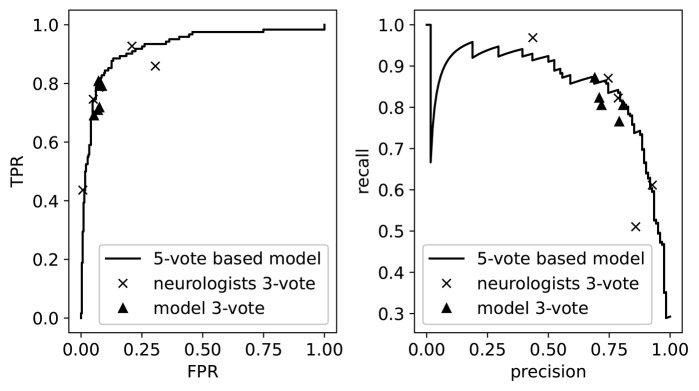
The receiver operating curve **(left)** and the precision-recall curve **(right)**. We also plot the performance of the neurologists and the model as measured on the 3-vote ground truths labels. TPR, True Positive Rate; FPR, False Positive Rate.

As our model outputs a continuous value, we can visualize its performance when varying the threshold for normal/abnormal. This is illustrated in Figure 4, where we show the precision-recall curve and the receiver operating curve. On top of these curves we plot the performances of the individual neurologists, as determined by their 3-vote score. These appear as points since the neurologists only assigned binary labels (i.e., they have a single threshold). We then see that these performances lie mostly on top of the model curve, which demonstrates once more the threshold assignment problem: varying the threshold of the model (i.e., traversing the curve) would allow us to mimic the performance of each of the neurologists. Also shown are the performances of the model on 5 of the 20 3-vote sets (one for each neurologist). By comparing the 3-vote scores, we see that the performance of our model is comparable to that of the experts.

### 3.5. Approximate Entropy: Interpretation of What It Measures

In this section, we explain and visualize what ApEn measures in a MEPTS. Technical details are given in the Supplementary Materials. A large ApEn (abnormal morphology) is found if the fluctuations have a steep slope, and/or if the fluctuations have a long duration. In terms used by neurologists: ApEn measures the duration of polyphasia, and the speed and height of the polyphasia (strength of dispersion).

**Figure 5:**
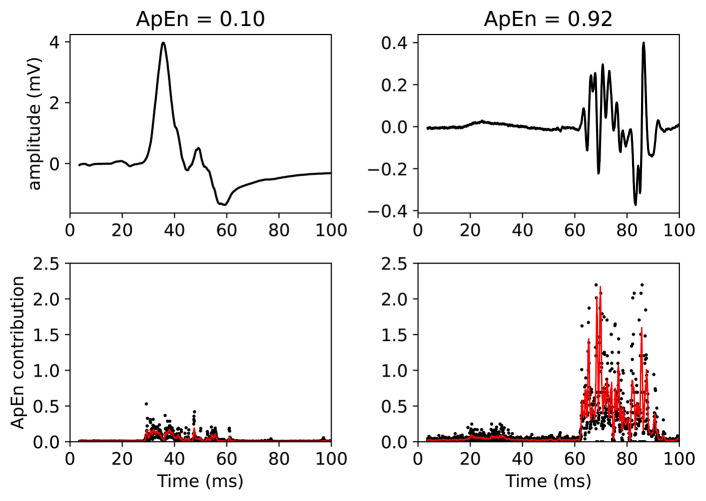
Example of two MEPTS (motor evoked potential time series) with low **(left)** and high **(right)** ApEn (approximate entropy). The time series are shown in the upper figures, while the ApEn contributions are shown in the lower figures. Each point of the time series has a corresponding ApEn contribution. A moving average of 11 points is also shown (red line). The final ApEn value is the average of all contributions. Note that the final ApEn value is normalized between 0 and 1.

This claim can be visually understood by plotting the MEPs and showing the associated ApEn contributions (see Figure 5). In this figure, two MEPs are compared. Each (sampled) point in the MEPTS leads to a contribution to the final ApEn value, which is found by averaging the individual contributions of all the points. To help with visual interpretation, a running average (red line) of the ApEn contributions is shown. Figure 5 contains a MEP with very low and very high ApEn. When the MEP is (almost) constant, the ApEn contribution is (almost) zero. When the MEP start moving up and down (i.e., fluctuating), the ApEn contribution becomes significantly higher than zero. To be more precise: a significant contribution is observed at the positions where the time series has a steep slope, as can be seen on the right side of Figure 5. When the movement is slow, the slope is shallow, and the ApEn contribution is low, as can be seen on the left side of Figure 5. A more detailed explanation, with more examples and details on how to implement the approximate entropy in practice, is provided in the Supplementary Materials.

One might be tempted to use simpler metrics than ApEn to quantify the abnormality of the morphology. A different metric could be counting the number of peaks (or, equivalently, the number of zero-line crossings) to count the number of phases (Nguyen et al., 2019). This works less well: the number of peaks feature has AUC = 0.76 on average on the 1-votes of the training set, compared to AUC = 0.92 for ApEn.

### 3.6. Approximate Entropy: Exploratory Clinical Implications

Given this new continuous score for the morphological abnormality of a MEPTS, we can now use it to annotate larger datasets without having to depend on neurologists to do this manually. To demonstrate this, we annotated a larger dataset of MEPs we have available from the RMSC, and explore some clinically relevant questions. The dataset is the same as in Yperman et al. (2020).

#### 3.6.1. Relation With Latency and Peak-to-Peak Amplitude

**Figure 6:**
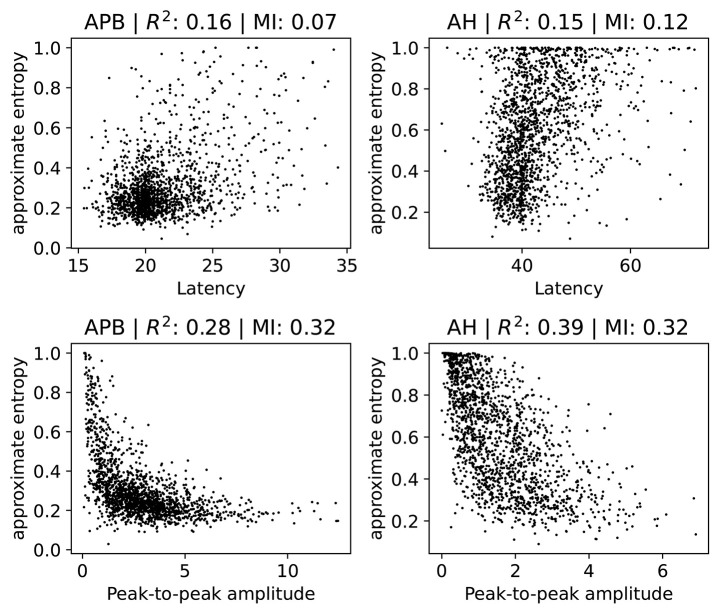
Scatter plots of the ApEn (approximate entropy) feature with the latency or peak-to-peak amplitude. We show separate plots for the hands (APB) and feet (AH) as the distributions are quite disparate. The titles of the plots indicate the muscles, as well as the *R*^2^ score and the mutual information (MI). A subset (2,000 randomly selected samples) of the complete dataset was used for visual clarity.

We investigate how the ApEn feature is related to the latency and the peak-to-peak amplitude. Note that low ApEn means normal morphology. Several scatter plots are shown in Figure 6, where we show separate plots for hands (AH) and feet (APB). For each of the scatter plots we also show the *R*^2^ measure and the mutual information (Cover and Thomas, 1991). We include the *R*^2^ measure mainly because it is often used in the literature, but we note here that it is of limited use in this case since not all relations are well-approximated by a linear dependence. Therefore, we also show the mutual information, which takes into account non-linear correlation as well. Intuitively, it measures how much knowing one of the variables reduces uncertainty about the other. It is equal to zero if two variables are independent, and higher values mean higher dependency.

There is no particularly strong relation between ApEn and latency. Both the *R*^2^ and the mutual information values are low in this case. Higher latencies are slightly correlated with higher ApEn, as evidenced by a linear fit which has a positive slope which differs from zero in a statistically significant manner. Generally speaking, abnormal morphology (which we quantify here using the approximate entropy) is an indication of demyelination, which also leads to higher latencies. Demyelination without changes to the morphology does occur, however, which explains the lack of a strong correlation. The observed small positive relation between ApEn and latency is therefore expected.

For the peak-to-peak amplitudes the correlation is higher than for the latencies, though still small. For both APB and AH, high ApEn is related to low peak-to-peak amplitude, and low ApEn is related to high peak-to-peak amplitude. This is also the expected behavior, as abnormal morphology indicates demyelination, which causes the motor response to be spread out over a longer time. This then leads to smaller amplitudes. The peak-to-peak amplitude is, however, also affected by purely axonal damage, which is separate from the demyelination and which does not affect the morphology as much. This agrees with the observed spread of the values in the plots. The extreme cases with values of the approximate entropy close to 0 and 1 are mostly artifacts, and are discussed in further detail in section 2 of the Supplementary Materials.

Finally, we note that a linear model that tries to estimate the approximate entropy using both the latency and the peak-to-peak amplitude obtains an *R*^2^ score of 0.4 and 0.31 for the legs and the arms, respectively.

These results show that the value of the ApEn is not fully determined by the latency and peak-to-peak values. It therefore contains information that is not captured by these two variables, indicating that it could be useful to include for clinical follow-up. ApEn could, e.g., be used as a variable in statistical models or for visualizing the evolution of a patient. Whether ApEn contains clinically relevant information that is not captured by the the latency and peak-to-peak amplitude requires further investigation.

#### 3.6.2. Relation With Disability

**Figure 7:**
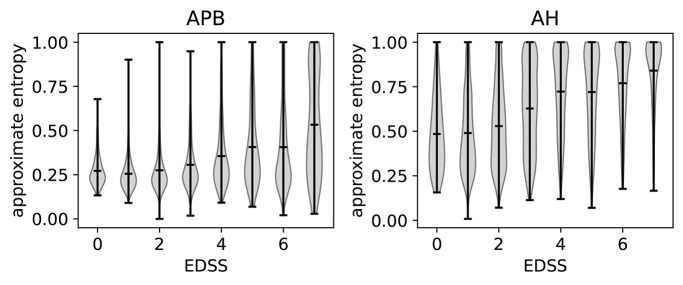
Violin plots to show the correlation between EDSS (expanded disability status scale) and the approximate entropy. We show separate plots for the hands (APB) and feet (AH) as the distributions are quite disparate. Time series with EDSS > 7.5 have been discarded as there are too few to be statistically relevant.

The relation of ApEn with disability can be investigated by using the expanded disability status scale (EDSS) (Kurtzke, 1983). A higher EDSS value indicates more disability. If an EDSS measurement is available one year before or after the MEP visit, the closest one is chosen as the EDSS of that visit. If none is available, the visit is not included in the analysis. In Figure 7, we show the violin plots to show the relation between the EDSS value and the ApEn for both the hands (APB) and the feet (AH). These plots show the mean and extremes of the samples (indicated by horizontal bars), as well as a rotated density plot showing the distribution of the samples.

There is a positive correlation between the EDSS and the ApEn, most clearly visible in the AH distributions. This shows that abnormal morphology is related to a higher amount of disability. We will discuss the results for the feet (AH) first, as these are the most sensitive for determining the EDSS value. This is because starting at an EDSS of 4, the tests only include the patient's ability to walk.

The ApEn distributions for AH show a transition toward higher values at EDSS = 3, which in a clinical practice is considered the onset of significant motor disability. Up to EDSS 4, the tests include evaluations of various parts of the central nervous system, so knowing the morphology of just the leg muscles will not contain enough information to determine the EDSS. This explains the spread of the distribution for those values. Starting at EDSS 4, however, the EDSS is based solely on the patient's ability to walk which is reflected in the fact that the distributions become more localized to high ApEn for higher EDSS values.

For APB (hands), there is little variation of the ApEn distribution between the EDSS values of 0 and 3. The distributions are more localized for these values than for those of 4 and up, as the arms are not directly considered for higher EDSS values. There is a slight increase of the average ApEn value between the EDSS values of 4–6. While low ApEn values are still the most common, there are now a significant number of high ApEn measurements (as can be seen from the density plots). Finally, for EDSS values higher than 6 we see a clear broadening of the distribution toward higher ApEn values, which translates into a higher average ApEn for EDSS > 6.

Comparing APB with AH, we note that the measurements performed on AH have, on average, a higher approximate entropy. This is to be expected as the asynchrony of the dispersed corticospinal volleys is accentuated by conduction in the peripheral nerve (Rossini et al., 2015). This effect is proportionate to the length of the peripheral nerve, which is higher for the AH muscle.

#### 3.6.3. Case Study: Predictive Ability of Approximate Entropy

In this section, we use the MEPs of patients that have 2-year follow-up data available (2,504 visits in total) to predict disability progression after 2 years using approximate entropy. These MEPs were also taken from the full retrospective dataset obtained from the RMSC, as in Yperman et al. (2020). A patient is said to have progressed if EDSS_*T*_1__ − EDSS_*T*_0__ >= 1.0 for EDSS_*T*_0__ ≤ 5.5, or if EDSS_*T*_1__ − EDSS_*T*_0__ >= 0.5 for EDSS_*T*_0__>5.5, as used by e.g., Goodkin et al. (1991). T_0_ is the time of the first measurement, and T_1_ is the time of the EDSS measurement between 1.5 and 3 years which is closest to the 2 year mark. An extensive investigation of this task for the MEP on this dataset was performed in Yperman et al. (2020).

**Figure 8:**
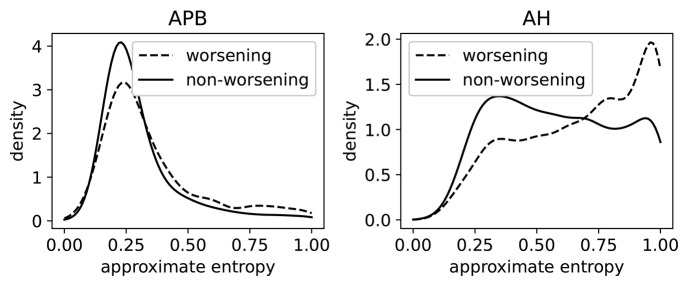
The distributions of the approximate entropy of the MEPs (motor evoked potentials) of the group of patients that have worsened after 2 years, and those that did not. Worsening is defined as disability progression as measured from the EDSS (expanded disability status scale), as discussed in section 3.6.3. This is done on a larger dataset (5,004 time series per limb) than the dataset that was rated. The distributions are made using a Gaussian kernel of width 0.05.

In Figure 8, we show the distributions of ApEn of both worsening and non-worsening patients. From this we see that the ApEn distributions in the hands (APB) are very similar. For the measurements made in the feet (AH), there is a clear difference between the two distributions: the patient population that is going to worsen is more skewed toward high ApEn (i.e., abnormal morphology). This shows that the morphology contains useful information regarding the task of predicting disability progression over 2 year's time.

A model based solely on the ApEn feature achieves an AUC of 0.62 on the prediction task. For comparison, the latency feature, found to have prognostic value in several works, obtains an AUC score of 0.67. We also tested whether adding the ApEn to a baseline prediction model based on the MEP latencies, EDSS, and age (this baseline model was used in Yperman et al., 2020) leads to improved prediction performance. We found, however, that this does not improve the performance for this task. The reason could be that the information in the ApEn is fully contained in the combination of MEP latencies, age, and EDSS. Another reason could be that the dataset is not large enough to pick up the additional information without overfitting. It could also be a combination of these two reasons. A larger MEP dataset would be required to obtain a clearer picture. Another interesting research direction is to look at the full longitudinal trajectories of MEP measurements to predict disability progression (De Brouwer et al., 2019), and investigate whether ApEn adds predictive performance for such an analysis.

## 4. Conclusion

Our results show that the approximate entropy feature (Pincus and Goldberger, 1994) can serve as a continuous score of the morphological abnormality of MEPs, removing the need for manual annotation by experts. Furthermore, it contains information not captured in the latency and peak-to-peak amplitude of the MEP, which are the variables most commonly used in statistical models. Having a valid proxy for morphological abnormality of MEPs has a number of advantages.

**Scalability:** Seeing as there is no longer a need for manual annotations, it is much easier to use the morphology feature in the analysis of much larger datasets. Annotating the 1,000 MEPTS used in this study took an average of 4 h when done manually. This quickly becomes a bottleneck for any data analysis pipeline. The analysis presented in section 3.6.3, which is based on data from a single center, would have taken upwards of 40 h to annotate manually. This becomes completely unfeasible when looking at multi-center datasets.

**Continuous:** ApEn is a continuous feature, with values ranging from 0 to 1 when normalized. This allows for a more nuanced interpretation, as opposed to the artificial dichotomization which often occurs due to practicality concerns. For example, prediction models would most likely benefit from being able to leverage this additional information.

**Reproducibility:** We see from our results that while the experts agree on the concept of morphology, there are still discrepancies in their choice of threshold of what constitutes a normal or an abnormal morphology. Datasets annotated by different experts would therefore be difficult to compare directly. Having a practical definition for morphological abnormality removes these inconsistencies.

**Predictive value:** We have shown in a cursory study that approximate entropy may be used as a predictor for MS disability progression. Whether it adds predictive power on top of other biomarkers (EP latency, MRI markers, neurofilament light chain, …) can now be investigated.

**Investigating clinical value:** We believe our results can support efforts to formulate recommendations and guidelines for the clinical use of MEPs in diagnosing and monitoring PwMS. For example, our morphology variable can be used to standardize EP morphologies from different measuring devices (or different centers). This standardized variable can then be used for clinical follow-up. Together with clinical findings and MR imaging, EP data may help us rationalize and optimize resources used in PwMS diagnosis and follow-up.

## Data Availability Statement

The original contributions presented in the study are publicly available. This data can be found here: https://github.com/JanYperman/deciphering-morphology.

## Ethics Statement

The studies involving human participants were reviewed and approved by Ethical commission of the University of Hasselt (CME2017/729). Written informed consent for participation was not required for this study in accordance with the national legislation and the institutional requirements.

## Author Contributions

JY performed the data analysis and developed the online labeling tool. JY, TB, DV, and LP decided on the data analysis methodology. NH, VP, and BV provided clinical feedback for the data analysis. MC, DD, GL, VP, and BV annotated the morphology of the evoked potential time series. LP coordinated the study. JY, TB, and LP wrote the original manuscript. All authors read and approved the final manuscript.

## Conflict of Interest

The authors declare that the research was conducted in the absence of any commercial or financial relationships that could be construed as a potential conflict of interest.

## Abbreviations

AH: abductor hallucis
AP: average precision
APB: abductor pollicis brevis
AUC/AUROC: area under the receiver operating characteristics curve
EDSS: expanded disability status scale
EP: evoked potential
EPTS: evoked potential time series
FPR: false positive rate
HCTSA: highly comparative time-series analysis
MEP: motor evoked potential
MEPTS: motor evoked potential time series
MRI: magnetic resonance imaging
MS: multiple sclerosis
PPMS: primary progressive MS
RMSC: revalidation and MS center
RRMS: relapsing-remitting MS
SD: standard deviation
SPMS: secondary progressive MS
TPR: true positive rate.
