# Supplementary material for: Deciphering the Morphology of Motor Evoked Potentials
Source: Front Neuroinform. 2020 Jul 14;14:28. doi: 10.3389/fninf.2020.00028 (PMC7381179; doi:10.3389/fninf.2020.00028)
Supplement: Supplementary file 1 [file Data_Sheet_1.PDF]

Supplementary Material

1 DISCUSSION TOP 10 FEATURES

The top 10 features for the individual neurologists are shown in Table S1. The names of the features are those provided by the HCTSA package. They can't be easily interpreted, but this isn't necessary to follow the discussion. Their implementation in HCTSA can be found at `https://github.com/benfulcher/hctsa/blob/master/Operations`. The first thing to note is that the top 10s are similar (i.e., the same features occur in the different top 10s), which points towards morphology being the same concept for all 5 neurologists. There are 2 features that occur in the top 10 of 4 neurologists: ApEn2_02 and CP_ML_StepDetect_l1pwc_10.rmsoff (see Table S1). ApEn2_02 measures the *approximate entropy* of the MEPTS. ApEn2_01 occurs in 3 of the top 10s and only differs from ApEn2_02 in a parameter choice (01 vs. 02). What these parameters mean is discussed in the Supplementary Material 2 and 3. This parameter difference only has a small influence on their values, so they are very similar. We find that each of the top 10s contains either ApEn2_01 or ApEn2_02. The third feature, CP_ML_StepDetect_l1pwc_10.rmsoff, has a high statistical correlation (-0.92 Pearson correlation) with the approximate entropy, but is conceptually different. There are several features that contain the word *sampen*. These features measure the sample entropy (with some variations in their implementation). Sample entropy is almost completely the same as approximate entropy, as discussed in Supplementary Material 2. So all the *sampen* features are conceptually related to the approximate entropy. There are more similarities between the top 10s. For example, ST_LocalExtrema_n25.meanabsext occurs in 3 top 10s, as does ST_LocalExtrema_n50.meanabsext.

2 APPROXIMATE ENTROPY: MATHEMATICAL DEFINITION

It is customary to claim that ApEn measures the *regularity* (Pincus et al., 1991) or *complexity* (Pincus, 1991) of a,time series . These two concepts are quite broad and therefore rather vague. Technical explanations often focus on the details of the algorithmic implementation (Pincus and Goldberger, 1994). We provide an explanation focused on its definition in terms of conditional probabilities. We conclude with more detailed technical comments.

We start by considering the following definition of ApEn (Pincus and Goldberger, 1994):

$$\text{ApEn} = -\left\langle \ln P(\text{pattern match at } m+1 | \text{pattern match at } m) \right\rangle_{\text{all patterns}}. \tag{S1}$$

To understand this definition, let us first focus on its most important part, the conditional probability:

$$P_m \equiv P(\text{pattern match at } m+1 | \text{pattern match at } m). \tag{S2}$$

What do we mean by a "pattern match at m"? The value of m determines the length of the pattern itself. In our case $m = 2$ or $m = 3$, so a pattern is any 2 or 3 subsequent values of the MEP. A pattern match is found if the same pattern occurs somewhere else in the MEP. Patterns don't need to be exactly the same to be classified as a pattern match. Rather, they should be roughly the same within some margin. The technical definition of a pattern match is given at the end of this section.

One can imagine going over all the patterns in the MEP, and tabulating all pattern matches. Note that each m subsequent values of the time series are a pattern, so going over all patterns means traversing the full time series from start to finish. We end up with a list of pattern matches for each pattern of length m.

We can write this as:

$$\text{pattern matches} : [p_{1,m}|p_{3,m}, p_{8,m}, p_{10,m}], [p_{2,m}|p_{4,m}, p_{5,m}], \ldots,$$

where the first pattern $p_{1,m}$ of length m is matched with patterns $p_{3,m}, p_{8,m},$ and $p_{10,m}$. The amount of pattern matches depends on the pattern.

We can do the same tabulation for pattern matches of length $m + 1$:

$$\text{pattern matches} : [p_{1,m+1}|p_{3,m+1}, p_{10,m+1}], [p_{2,m+1}|p_{4,m+1}], \ldots$$

Here, $p_{1,m+1}$ is the first pattern of length $m + 1$. Note that this is just $p_{1,m}$ with the next time series value included.

We are now able to calculate the probability of a pattern match at length $m + 1$, *conditional* on there already being a pattern match at length m. For example, for $p_{1,m}$ there are 3 pattern matches, of which 2 remain a match for $p_{1,m+1}$. This translates to the following conditional probability

$$P(\text{pattern match at } m + 1|\text{pattern match at } m) = 2/3 \tag{S3}$$

for pattern $p_{1,m}$. Similarly, for $p_{2,m}$ we find

$$P(\text{pattern match at } m + 1|\text{pattern match at } m) = 1/2. \tag{S4}$$

If patterns are predictable at length m, this conditional probability is high, and vice versa. Time series with highly predictable patterns can be seen as highly regular, or of low complexity.

To calculate the ApEn value (Eq. S1), we take the logarithm $\ln(\cdot)$ of the conditional probability P_m (Eq. S2). We do this because we are interested in the order of magnitude of the conditional probability: $P_m = 10^{-3}$ versus $P_m = 10^{-1}$ is transformed to -3 versus -1 (at least approximately, since we take $\ln(\cdot)$ and not $\log_{10}(\cdot)$). Highly regular MEPs ($P_m \approx 1 = e^0$) are mapped to almost zero, while very irregular MEPs ($P_m \approx 0 = e^{-\infty}$) are mapped to $-\infty$.

After taking the logarithm of P_m, we average over all patterns in the MEP, hence we write $\langle\cdot\rangle_{\text{all patterns}}$ in Eq. S1. Averaging means going over all patterns of the time series from start to finish, summing all $\ln(P_m)$ values, and dividing them by the number of patterns.

Finally, we take minus this value to ensure that highly regular MEPs have low ApEn (close to zero), and irregular MEPs have high ApEn (high positive values). We further normalize the ApEn values, so that they all lie between 0 and 1, see Supplementary Materials 4.

Besides m, there is a second parameter for ApEn, namely r. This parameter determines the distance threshold for confirming that two patterns are considered a match. For $m = 2$, values of r between 0.1 and 0.25 times the standard deviation of the time series are advised (Pincus and Goldberger, 1994). We have $r = 0.2$ times the standard deviation, which is within the normal range.

To conclude, ApEn(m=2, r=0.2) (denoted as ApEn2_02 in Table S1) is good at reproducing binary morphology classification of MEPs. It measures the regularity of the time series , by which we mean the predictability of patterns of length m.

For completeness, we provide some further technical notes. Strictly speaking, the algorithmic implementation of ApEn only converges to Eq. S1 in the limit of time series length $N \rightarrow \infty$. For $N = 1920$ this effect is negligible. The definition of a pattern match depends on the distance measure between two patterns. For a time series consisting of N data points $\{u(1), \ldots, u(N)\}$, a sequence (or pattern) of length m is defined as $\mathbf{x}(i) = [u(i), \ldots, u(i+m-1)]$, with $i \in [1, N-m+1]$. The distance $d(\mathbf{x}(i), \mathbf{x}(j))$ between two patterns is defined as

$$d(\mathbf{x}(i), \mathbf{x}(j)) = \max\left(|u(i+k) - u(j+k)|\right), \forall k \in [0, m-1]. \tag{S5}$$

In words: the distance $d(\mathbf{x}(i), \mathbf{x}(j))$ between two patterns is the maximum distance between their respective elements.

From the definition of the distance threshold we notice two measurement artifacts of the MEPs that influence the ApEn calculation. Of the few MEPs that have very low ApEn $\in [0, 0.1]$, most are artificially low because the time series wanders away from the baseline at the end. This baseline wander leads to an artificially large standard deviation, which makes the pattern match threshold, $r = 0.2 \times$ standard deviation, artificially high. As a result, pattern matches are easy to obtain, and ApEn is underestimated. On the other end, quite some MEPs with high ApEn $\in [0.9, 1.0]$ are influenced by measurements noise. If the amplitude is low, r is also low. For very low amplitudes, this means that measurement noise can lead to not passing the pattern match threshold. This artificially overestimates the ApEn. These issues are visualized in Figure S1.

The sample entropy removes a small bias in the definition of ApEn (Richman and Moorman, 2000). This bias occurs because ApEn counts the pattern match with itself as a match. Regularity is therefore systematically overestimated. Sample entropy does not include the match of a pattern with itself when calculating P_m. This issues is, however, of little practical importance for the time series considered here.

3 APPROXIMATE ENTROPY: FURTHER INTERPRETATION OF WHAT IT MEASURES

We expand on the explanation given in Section 3.5. Figures S2 and S3 further show the behaviour of ApEn for different MEPTS. One can observe that, besides measuring the strength of the polyphasia, the ApEn is also influenced by the duration of the polyphasia. An example is shown in Figure S2. When fluctuating, the size of the ApEn contribution is similar for both MEPs (around 0.5). Nevertheless, the MEP on the right fluctuates for a longer time (between 55 ms and 80 ms) than the one on the left (between 25 and 35 ms). Therefore, the ApEn value on the right is higher (0.53) compared to the one on the left (0.20). Figure S3 shows two MEPs with similar ApEn values, but with different contributions: on the left the duration of fluctuations is short but with high strength, while on the left the duration is longer but their strength is lower.

As noted in the main text, other simpler metrics to quantify the abnormality of the morphology, such as the number of peaks, work less well. This is shown in Table S1 (AUC = 0.76 on average on the 1-votes of the training set). Other simple features, such standard deviation (AUC = 0.82 on average on the 1-votes of the training set), also don't work as well as ApEn (AUC = 0.92).

4 APPROXIMATE ENTROPY: PRACTICAL IMPLEMENTATION

For readers who wish to use this feature in practice on their own MEPTS datasets, we provide here a brief overview on the procedure to calculate the approximate entropy feature. We implemented the steps outlined

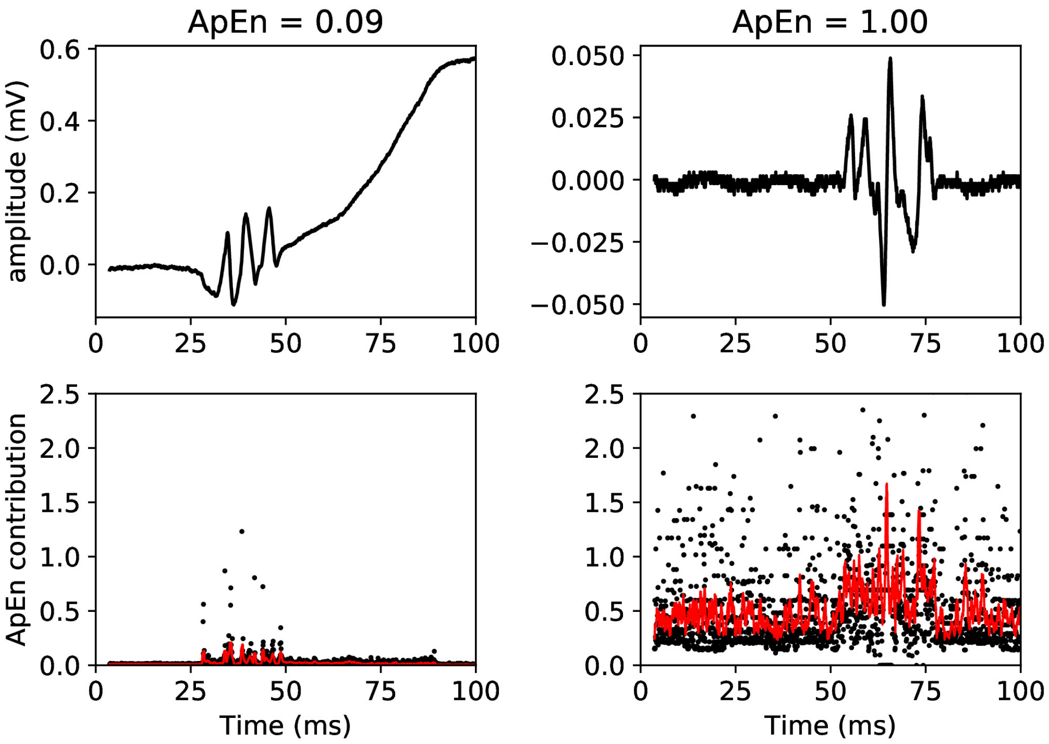

Figure S1. Example of two MEPTS with extremely low (left) and high (right) ApEn values. The time series are shown in the upper figures, while the ApEn contributions are shown in the lower figures. Each point in the time series has a corresponding ApEn contribution. A moving average of 11 points is also shown (red line). Note that the final ApEn value is normalized between 0 and 1. Both values are caused by anomalies in the measurement. The ApEn for the MEP on the left is too low because of baseline wander, while the one on the right is too high because of measurement noise.

in this section in a Python script (**calculate_apen.py**) which is available on the github repository of this paper (`https://github.com/JanYperman/deciphering-morphology`).

- Make sure the duration of the measurement is 100ms.
- The sampling rate should be 19.2 kHz, resulting in 1920 samples per time series . In case the sampling rate is not this value, resample the time series to obtain 1920 samples per time series .
- Remove the first 70 points, as this will usually include an artifact peak.
- Use the code provided by the HCTSA library to calculate the ApEn feature.[1].
- Use the formula
$$\hat{\mathbf{f}} = \left\{1 + \exp\left[-\frac{\mathbf{f} - \mathrm{median}(\mathbf{f})}{\mathrm{iqr}(\mathbf{f})/1.35}\right]\right\}^{-1}, \tag{S6}$$
where $\mathrm{iqr}(\mathbf{f}) = 0.06292$ and $\mathrm{median}(\mathbf{f}) = 0.06603$ to match the normalization we used.
- Rescale between 0 and 1 using:
$$\frac{\mathbf{f} - \min(\mathbf{f})}{\max(\mathbf{f}) - \min(\mathbf{f})} \tag{S7}$$
where $\min(\mathbf{f}) = 0.2302$ and $\max(\mathbf{f}) = 1.$ to match our normalization.

[1] `https://github.com/benfulcher/hctsa/blob/master/Operations/EN_ApEn.m`

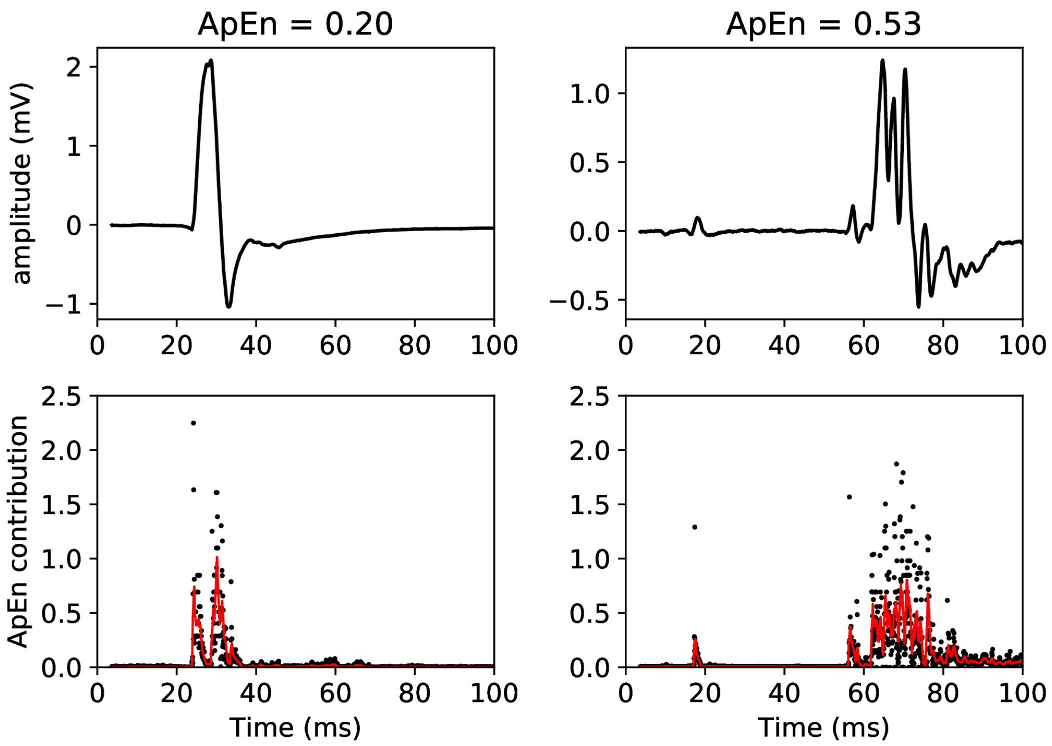

Figure S2. Example of two MEPTS with low (left) and average (middle) ApEn values. The time series are shown in the upper figures, while the ApEn contributions are shown in the lower figures. Each point of the time series has a corresponding ApEn contribution. A moving average of 11 points is also shown (red line). The final ApEn value is the average of all contributions. Note that the final ApEn value is normalized between 0 and 1.

5 COHEN'S KAPPA

In this section we illustrate why the Cohen's κ metric should be used alongside simple percentages of agreement. The metric that seems most natural to consider is simply the percentage of labels where the raters agree. This metric, however, is greatly influenced by class imbalance. To illustrate, consider the case where 100 samples are annotated. If each neurologist were to randomly pick 10 different samples to label as abnormal, there would still be an agreement fraction of about 82%, even though they do not agree on what abnormality is at all. This happens because the normal case occurs more frequently than the abnormal case. For Cohen's κ the score for this example would be 0.

6 ONLINE LABELING TOOL

In this section we provide a few technical details of the online labeling tool. We used the Django web framework, mainly because it is based on Python and therefore allowed seamless integration with our data analysis pipeline. Each neurologist received a unique username and password to ensure independent labeling, and to avoid access to the data by unauthorized users. The dataset was stripped to just the time series and a unique identifier of the visits before being uploaded to the web server so as to ensure privacy. The labels were stored in a relational database (sqlite3 backend). We needed the neurologists to be able to fully inspect the time series including panning, zooming, hiding time series etc. We implemented this functionality using the mpld3-library, which allows converting matplotlib graphs to HTML (including JavaScript to handle the interactions) which can then be run in the browser. In particular, we used the InteractiveLegendPlugin and the MousePosition plugins to allow the hiding of certain time series and

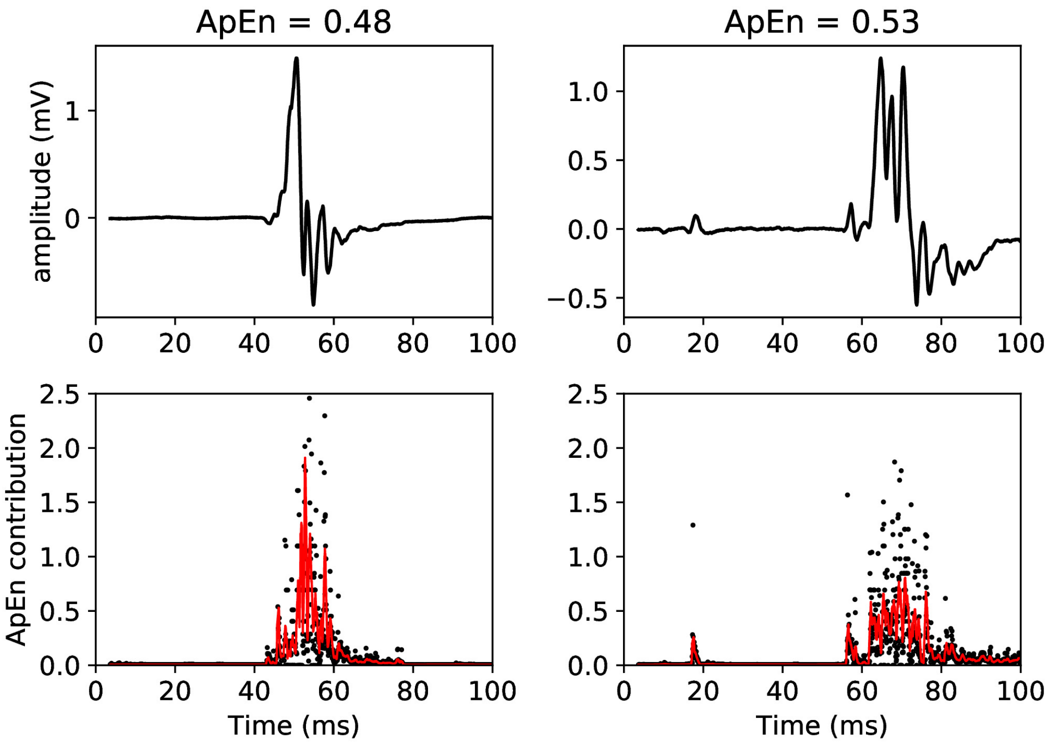

Figure S3. Example of two MEPTS, both with average ApEn values. The time series are shown in the upper figures, while the ApEn contributions are shown in the lower figures. Each point of the time series has a corresponding ApEn contribution. A moving average of 11 points is also shown (red line). The final ApEn value is the average of all contributions. Note that the final ApEn value is normalized between 0 and 1.

displaying the mouse coordinates respectively. The website was hosted on pythonanywhere.com, which provides out-of-the-box support for Django. The dataset was stripped to just the time series and a unique identifier of the visits before being uploaded to the pythonanywhere.com servers so as to ensure privacy.

7 FILTER IMPACT

As discussed in section 2.2, the timeseries were gathered from two separate machines at the clinic in Pelt. One machine applied a bandpass filter between 0.6Hz and 10kHz. We will refer to this as machine A. The other machine applies a 100 Hz high-pass filter to the timeseries. We will refer to this as machine B. In this section we study the impact of the 100 Hz high-pass filter on the results.

After removing doubles and timeseries that are labeled to be bad data, we have 732 time series of machine A, and 101 of machine B. We use cross-validation (1000 random splits, each ensuring the same patient does not occur in both the train and test set) on the entire dataset to assess the performance of the approximate entropy on each. The results are shown in Table S2, labeled as A [NF] and B for machine A and machine B respectively. Both machines perform well, with machine B outperforming machine A on all metrics. From this we can conclude that the approximate entropy feature works well for both machines. At least for the morphology, it seems that the high-pass filter helps to get more consistent results. Both the results of the model and those of the neurologists significantly improve. To illustrate the separability of the samples in the dataset, based on their approximate entropy, we show a scatter plot in Figure S4. The horizontal axis only serves to space out the markers for visual clarity. The vertical axis shows the approximate entropy. The plots demonstrate that for the filtered time series the

samples are almost perfectly separable. For the time series that have not been filtered there is significantly more overlap. For the filtered time series there are multiple features that result in a good separation, but they do not seem to be as robust to the presence of the lower frequencies as the approximate entropy feature.

To check the impact of the filter on the approximate entropy, we applied a 100 Hz highpass butterworth filter of order 1 to the timeseries of machine A before calculating the approximate entropy, after which we run the same pipeline again. The results of this are also shown in S2, labeled as [F]. Note that we did not run these filtered timeseries by the neurologists again, so that column has no values. It seems that the low frequencies have little impact on the performance of the approximate entropy feature. The difference in performance between both machines is then likely caused by other factors. It is possible that the experts rate the unfiltered timeseries less consistently, which then introduces noise in the ground truths labels, ultimately leading to a lower overall performance. The difference could also be related to another difference in set-up between the two machines. We are comparing the two separate machines under the assumption that the main difference between them is the filtering of the measurements. This is a reasonable assumption, but there could be other variables that may be causing this effect. The goal of this work, however, was to show that approximate entropy is a valid proxy for morphological abnormality, which these results show to be true for both machines.

The results of this section may be reproduced by using the *generate_supplementary.py* script in the repo of this work. (`https://github.com/JanYperman/deciphering-morphology`)

REFERENCES

Pincus SM, Gladstone IM, Ehrenkranz RA. A regularity statistic for medical data analysis. *Journal of Clinical Monitoring* **7** (1991) 335–345. doi:10.1007/BF01619355.

Pincus SM. Approximate entropy as a measure of system complexity. *Proceedings of the National Academy of Sciences* **88** (1991) 2297–2301. doi:10.1073/pnas.88.6.2297.

Pincus SM, Goldberger AL. Physiological time-series analysis: what does regularity quantify? *American Journal of Physiology-Heart and Circulatory Physiology* **266** (1994) H1643–H1656.

Richman JS, Moorman JR. Physiological time-series analysis using approximate entropy and sample entropy. *American Journal of Physiology-Heart and Circulatory Physiology* **278** (2000) H2039–H2049. doi:10.1152/ajpheart.2000.278.6.H2039. PMID: 10843903.

Nguyen D, Rissanen S, Julkunen P, Kallioniemi E, Karjalainen P. Principal component regression on motor evoked potential in single-pulse transcranial magnetic stimulation. *IEEE Transactions on Neural Systems and Rehabilitation Engineering* **27** (2019) 1521–1528.

Rank N1	AUC N1	Feature N1	Rank N2	AUC N2	Feature N2
1	0.940	ST_LocalExtrema_n25.meanabsext	1	0.934	CP_ML_StepDetect_l1pwc_10.rmsoff
2	0.940	CP_ML_StepDetect_l1pwc_10.rmsoff	2	0.931	SY_SpreadRandomLocal_200_100.meansampen1_015
3	0.937	CO_Embed2_Dist_tau.d_iqr	3	0.931	CO_Embed2_Dist_tau.d_iqr
4	0.936	**ApEn2_02**	4	0.929	SY_SlidingWindow_sampen_ent5_10
5	0.934	ST_LocalExtrema_l100.meanabsext	5	0.928	SY_SpreadRandomLocal_ac2_100.meansampen1_015
6	0.933	SY_SpreadRandomLocal_200_100.meansampen1_015	6	0.927	**ApEn2_02**
7	0.932	SY_SlidingWindow_sampen_ent5_10	7	0.927	**ApEn2_01**
8	0.932	SY_SpreadRandomLocal_ac2_100.meansampen1_015	8	0.926	EN_CID.CE1_norm
9	0.932	ST_LocalExtrema_n50.meanabsext	9	0.925	EX_MovingThreshold_1_002.meanqover
10	0.931	ST_LocalExtrema_l50.meanabsext	10	0.921	ST_LocalExtrema_n25.meanabsext
1688	0.736	Number of peaks	1379	0.753	Number of peaks

Rank N3	AUC N3	Feature N3	Rank N4	AUC N4	Feature N4
1	0.958	SY_SpreadRandomLocal_200_100.meansampen1_015	1	0.947	CP_ML_StepDetect_l1pwc_10.rmsoff
2	0.957	SY_SpreadRandomLocal_ac2_100.meansampen1_015	2	0.943	PH_Walker_prop_01.sw_meanabsdiff
3	0.953	**ApEn2_01**	3	0.942	ST_LocalExtrema_n50.meanmax
4	0.953	**ApEn2_02**	4	0.940	FC_LocalSimple_median7.meanabserr
5	0.951	CP_ML_StepDetect_l1pwc_10.rmsoff	5	0.939	**ApEn2_02**
6	0.949	FC_LocalSimple_median5.meanabserr	6	0.938	ST_LocalExtrema_l50.meanmax
7	0.948	FC_LocalSimple_median7.meanabserr	7	0.937	PH_Walker_biasprop_05_01.sw_meanabsdiff
8	0.948	FC_LocalSimple_mean4.meanabserr	8	0.937	FC_LocalSimple_median5.meanabserr
9	0.948	FC_LocalSimple_mean3.meanabserr	9	0.937	ST_LocalExtrema_n100.meanmax
10	0.948	PH_Walker_prop_05.sw_meanabsdiff	10	0.935	FC_LocalSimple_mean4.meanabserr
1334	0.776	Number of peaks	1049	0.788	Number of peaks

Rank N5	AUC N5	Feature N5
1	0.874	ST_LocalExtrema_n50.meanabsext
2	0.872	ST_LocalExtrema_l50.meanabsext
3	0.869	ST_LocalExtrema_n25.meanabsext
4	0.868	EX_MovingThreshold_01_002.pkick
5	0.868	EN_CID.CE1_norm
6	0.862	**ApEn2_01**
7	0.861	EX_MovingThreshold_01_002.meanq
8	0.855	ST_LocalExtrema_n100.meanabsext
9	0.853	SP_Summaries_pgram_hamm.numPromPeaks_1
10	0.852	CO_AddNoise_1_quantiles_10.ac2
1178	0.723	Number of peaks

Table S1. The individual top 10 features of the neurologists. The order is determined by the average performance (AUC) of the features on the 1-vote labels, from a 3-fold cross-validation on the training set. The type of feature that was ultimately chosen, approximate entropy, is shown in bold. The names of the features are those provided by the HCTSA package. We also include the number of peaks feature (not implemented in HCTSA) for reference. This variable seemed a good handpicked choice after the teleconference discussions on the definition of morphological abnormality, see also, e.g., (Nguyen et al., 2019)

Rater type	model [3-vote] (std) A [F]	A [NF]	B	model [5-vote] (std) A [F]	A [NF]	B	neurologists [3-vote] (std) A [F]	A [NF]	B
**AUC**	0.93 (0.02)	0.92 (0.02)	0.97 (0.02)	0.93 (0.02)	0.92 (0.03)	0.99 (0.02)	-	N/A	N/A
**AP**	0.79 (0.06)	0.80 (0.05)	0.96 (0.05)	0.81 (0.07)	0.81 (0.06)	0.99 (0.02)	-	N/A	N/A
**F1**	0.72 (0.05)	0.74 (0.05)	0.91 (0.06)	0.74 (0.06)	0.76 (0.06)	0.94 (0.06)	-	0.69 (0.05)	0.83 (0.09)
**Accuracy**	0.86 (0.03)	0.87 (0.02)	0.93 (0.04)	0.87 (0.03)	0.88 (0.03)	0.96 (0.04)	-	0.83 (0.03)	0.88 (0.06)
**Precision**	0.74 (0.06)	0.76 (0.06)	0.92 (0.07)	0.75 (0.07)	0.76 (0.08)	0.95 (0.08)	-	0.74 (0.03)	0.86 (0.07)
**Recall**	0.72 (0.07)	0.74 (0.07)	0.91 (0.08)	0.74 (0.07)	0.76 (0.08)	0.94 (0.08)	-	0.74 (0.03)	0.85 (0.07)
**Cohen**	0.63 (0.06)	0.66 (0.06)	0.85 (0.08)	0.66 (0.07)	0.68 (0.07)	0.90 (0.09)	-	0.58 (0.05)	0.74 (0.12)

Table S2. Comparison of the cross-validation performance of the approximate entropy on the timeseries from machine A and B. The performance on machine A with and without 100Hz high-pass filtering is denoted by [F] and [NF], respectively. The values shown in brackets are the standard deviations of the average performance. Note that for these metrics the *abnormal* class was used as the positive label. Note that there are no values for the filtered time series (F) for the neurologists. Abbreviations used: Area Under the receiver operating characteristic Curve (AUC), Average Precision (AP).

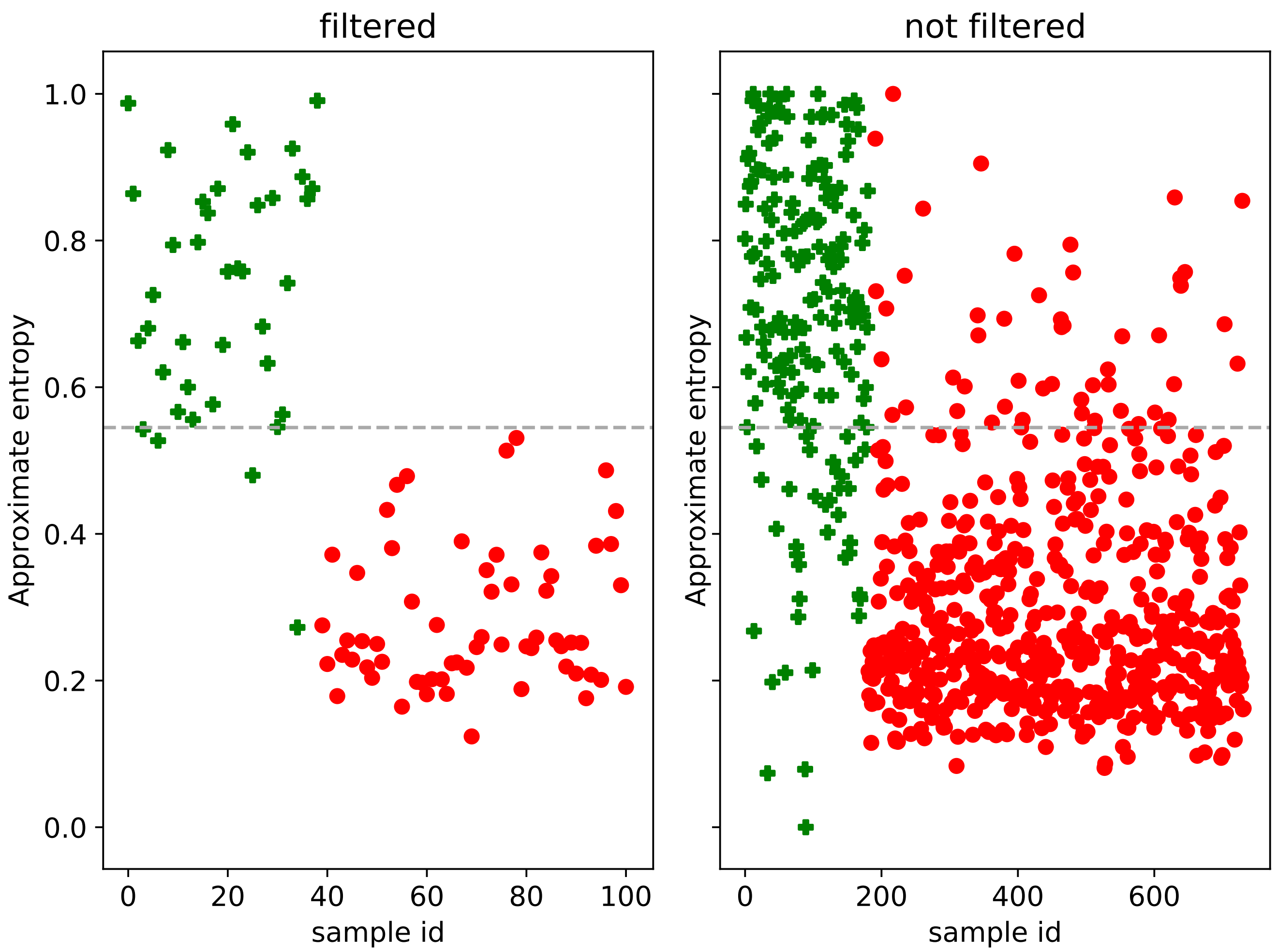

Figure S4. Approximate entropy scatter plot to illustrate separability for both machines. The grey dashed line indicates the threshold that was chosen in the paper. The plusses and circles indicate the abnormal morphologies and the normal morphologies respectively (as determined by the 5-vote of the neurologists). The horizontal axis serves only to increase spacing between the individual samples to increase visual clarity.
